# The meaning of the MS Degree in Medical Physics, Part 3

**DOI:** 10.1120/jacmp.v15i3.5011

**Published:** 2014-05-08

**Authors:** Michael D. Mills

To continue the theme of my previous two editorials, I thought I would open a discussion on where we could find some common ground and workable solutions to the problems mentioned therein. In other words, I hope to meddle with the status quo! I trust most of you sense both the importance and urgency for the community to develop a consensus as to how these problems are conceived. To recapitulate:

Upon completion of a CAMPEP Master's degree in medical physics, 10%−15% of the graduates pursue doctoral degrees; most of the rest elect to compete for a CAMPEP residency program. The primary drawback of choosing medical physics as a career and matriculating in a CAMPEP MS program is that, currently, only about one in four MS graduates successfully compete for a CAMPEP residency slot, which is required for entry into the profession. Also, few MS programs provide free tuition and/or a stipend. This means that the typical MS student who wishes to have a clinical career will need to commit to borrowing up to $40,000 to pay for the CAMPEP MS with only a small (about one in four) chance of entering the profession as a Boarded Professional (Qualified) Medical Physicist. The cost is similar to other medical professional degree programs, but the difference is students are guaranteed entry into the profession upon successful completion of the program. The question of whether this cost is too high I will leave for another editorial.

I submitted this was unfair to the student, and that we would lose many excellent potential colleagues to other professions unless this situation was corrected. It is true that a clinical career does not suit everyone, and some medical physics MS graduates would be more interested in other opportunities. Removing those that elect to earn a PhD and those not interested in a clinical career from the pool of residency competition still means that, at best, fewer than one in three MS graduates will successfully complete a CAMPEP residency and enter the profession.

While CAMPEP does have restrictions for the number of residents that may enter an accredited residency program at an institution (based on funding and the number of faculty and staff), there are no limits on the number of students a CAMPEP MS program may accept. Nor does CAMPEP's mandate currently extend to implementing such limits. Consider that increasing the number of residency slots requires both a proportionally increased effort on the part of existing faculty and staff, along with commensurate funding for any additional positions. Conversely, for academic MS programs, especially those that offer limited clinical exposure or experience, there is only a small increment of administrative effort required to accommodate additional students. Additionally, there is no policy disincentive for academic institutions to create new CAMPEP MS programs or expand existing ones. There are financial incentives for these programs to grow in size and number; in some cases additional tuition revenues can be used to compensate faculty for their teaching efforts, as well as provide some support for research activities that may not otherwise be funded.

The modern medical school curriculum distributes a breadth of clinical experiences throughout the four‐year program. Medical specialty training is received in the physician's residency program. Medical physics programs mostly separate the classroom (academic MS) from the clinical (CAMPEP residency) experience. Some CAMPEP MS programs are being encouraged to reduce introductory clinical medical physics experience and increase research efforts, assuming all clinical components are now covered during the medical physicist's residency program's specialty (radiation therapy or imaging). Conversely, others are looking to add introductory clinical medical physics experiences to increase their graduates' chances of landing a CAMPEP residency. Unless CAMPEP MS programs add many more documented clinical experiences (requiring significant faculty and staff commitments), it would be highly unlikely CAMPEP would ever consider limiting class sizes for any accredited academic program. While almost every medical and allied health profession specifies limits on the class sizes and residency positions, to date this has not been an issue to consider respecting CAMPEP accreditation for academic programs. So where should such restrictions come from, if anywhere? Is there any alternative? Should our profession just continue to accredit new programs and build even larger programs every year with impunity? As I see it, there is simply not the political will within the universities or the AAPM to oppose the financial incentives of some MS program directors.

Some would suggest that this is a supply and demand issue that will stabilize within a few years. Yes, several supply and demand economies govern the medical physics entry process, including competition for academic positions, residency positions, and employment. But is this an easy way to excuse the troubling ethics of asking MS students to borrow money to support institutional research programs (resulting in publications that support faculty promotion and tenure), knowing that the majority of these students will be denied entry to the profession? Some non‐board‐certified medical physicists are being employed as technicians or assistants in the US and Canada, and there may be a trend to eliminate qualified medical physicist positions in favor of these positions. I conclude there may be a conflict of job security interests between the town and gown of the medical physics community.

The number of graduate programs seeking accreditation has fallen recently. With the introduction of the ABR requirement, CAMPEP saw an increase in applications of six to seven per year for 2009, 2010, and 2011, but the number dropped to four for 2012 and 2013, and for 2014, to date, CAMPEP has received two applications. Still, four new programs a year is probably six too many for our current supply/demand economy.

So we have two issues: 1) creating too many MS physicists for the jobs available (there is the reality that the many CAMPEP MS graduates who cannot enter a residency could undermine the job security and quality of life of QMPs), and 2) not being fair to students facing both the potential of substantial debt along with a small chance of entering the profession. Do we really want a two‐tiered profession, like Health Physics, where only a fraction of members hold professional board certification? Is that where we are headed with the current incentives in place?

Some would argue this is no different from graduate programs found in the fine arts, social sciences, etc., which students undertake with no guarantee of a job in the field at the end of the program. I believe the situation for medical physics to be conceptually different, as it is not a job but a CAMPEP residency program and entry to the profession that is more likely not to be obtained than otherwise. The fine art and social science program graduates have the degrees that admit them to entry‐level jobs; the unsuccessful CAMPEP MS graduate cannot enter the profession and get a job. The MS graduate may compete for the physics assistant positions, and yes, such a position may pay even more than a corresponding position in the fine arts and social sciences, but that was not the reason the student borrowed the money to enter the program.

Please consider reviewing the following article published in a previous JACMP issue, *Medical Physics Residency Consortium: collaborative endeavors to meet the ABR 2014 certification requirements*, by Brent C. Parker and colleagues.^(1)^ The remarkable aspect of this CAMPEP MS program at Louisiana State University (LSU) is through its partnership with Mary Bird Perkins Cancer Center (MBPCC), it provides opportunity for a resident position to every MS student who enters the program upon acceptance. This decision, along with providing free tuition and stipend, should put LSU at the top of the application list of many seeking to enter the medical physics profession, as it greatly reduces the risk of entering the field. I hope other programs will be moved to follow suit and offer similar programs, as it is the best answer I have seen to the fairness question raised above.

Could CAMPEP do something to encourage other MS programs to likewise offer a guaranteed residency slot to selected applicants to their MS program? CAMPEP currently requires a lot of information to be posted on their website. Could these information items be offered as well?

- To the Academic Program Director: Does your program immediately (at matriculation) guarantee any CAMPEP Residency Positions to graduates of the Academic Program? If so, to all students, or just to some? If to some, how many residency positions and what was the total number in your graduating academic class?
- The following questions are included in the required annual report and should be available on the academic program's website: Are residency positions (if any) offered to the student in competition with other students in the same graduating class? If so, how many positions are offered and what is the class size respecting your current graduating class? What are the criteria of selection for those students admitted to the residency program?

The statistics available on program graduates are published annually by the SDAMPP on its website. While this does not address the overall problem of overproduction of CAMPEP MS graduates, it would create some level of greater fairness for the students. If a student can obtain a guaranteed for a reserved residency position at (CAMPEP MS Academic Program) matriculation following a successful application, that would be a desirable outcome. I sincerely hope the LSU/MBPCC initiative challenges other schools to follow with similar programs. Schools that offer the residency at the beginning of MS matriculation should be able to compete for the best students, and prospective students should have the right to know where these options exist. At the moment this is rare, probably because graduate programs and residency programs are funded from different sources. Only a small percentage of the accredited graduate programs have strong links with residency programs and few of those are linked financially.

If a school chooses to offer one or two guaranteed residencies to their best incoming student(s), the other students would still be able to compete for other CAMPEP residencies, perhaps in the same institution and perhaps in others. Finally, if a CAMPEP academic MS program chooses to wait until the program is 18 months completed before identifying the student(s) who is/are selected for the CAMPEP residency, it would be helpful to have the selection criteria specified on the website. Especially if factors other than GPA are considered in the evaluation, these factors should be specified before the student begins the program. For example, if a student with an alternative physics PhD decides to enroll in a CAMPEP MS program, would that student be preferred for an open residency slot above the top MS student despite having a lower GPA? If that is the case, it seems only fair to share that information up front.

An incoming student facing enormous debt should have the maximum information possible. This means: Where can the student find those programs that fund their graduate students and/or guarantee opportunity for a reserved residency position? If the student cannot get such a guarantee, what does he/she need to do to qualify for one of the in‐house residency positions that would be available two years from now? Would this be a problem for CAMPEP to require programs to post such information? Perhaps the answers will support and affirm the dignity of our students while we collectively define the meaning of the MS degree in Medical Physics.

I want to thank Brenda Clark for her contribution of insightful thoughts and comments on this editorial while completely exonerating her for its meddling content, which is exclusively my own.
